# Stage migration and survival outcomes in patients with cervical cancer at Stage IIIC according to the 2018 FIGO staging system: a systematic review and meta-analysis

**DOI:** 10.3389/fonc.2024.1460543

**Published:** 2024-10-01

**Authors:** Ling Han, Yali Chen, Ai Zheng, Xin Tan, Hengxi Chen

**Affiliations:** ^1^ Department of Obstetrics and Gynecology, West China Second University Hospital, Sichuan University, Chengdu, Sichuan, China; ^2^ Key Laboratory of Birth Defects and Related Diseases of Women and Children (Sichuan University), Ministry of Education, Chengdu, Sichuan, China; ^3^ Day Surgery Department, West China Second University Hospital, Sichuan University, Chengdu, Sichuan, China

**Keywords:** cervical cancer, stage migration, survival outcome, systematic review, meta-analysis

## Abstract

**Objective:**

To summarize stage migration and survival outcomes in patients with cervical cancer at Stage IIIC according to the 2018 FIGO staging system, and to investigate prognostic factors influencing Stage IIIC1.

**Methods:**

PubMed, Embase, Cochrane Central Register of Controlled Trials (CENTRAL), International Clinical Trials Registry Platform (ICTRP), and Clinical Trials.gov were searched from inception to February 6, 2024. The analysis was conducted using STATA 16.0.

**Results:**

A total of 25 studies with 82954 cervical cancer patients were included in the analysis. The migration rates to FIGO 2018 Stage IIIC ranged from 18% to 37% for early-stage tumors (Stage IB to IIA) in FIGO 2009, and from 32% to 52% for advanced stage tumors (Stage IIB to IIIB). The overall survival (OS) for Stage IIIC1 is poorer compared to Stage IB1 (HR 0.53, 95% CI 0.35-0.80, p=0.003) and Stage IB2 (HR 0.61, 95% CI 0.43-0.85, p=0.004). It is comparable to Stage IB3, yet it shows better survival outcomes than Stages IIB (HR 2.91, 95% CI 1.01-8.39, p=0.047), IIIA (HR 1.96, 95% CI 1.78-2.17, p=0.000), and IIIB (HR 1.56, 95% CI 1.04-2.35, p=0.031). Tumors size ≥4cm (HR 1.45, 95% CI 1.10-1.92, p=0.00), metastatic lymph node ≥ 3 (HR 2.21, 95% CI 1.56-3.15, p=0.000) and T stage are prognostic factors for OS of Stage IIIC1.

**Conclusions:**

The migration rates to FIGO 2018 Stage IIIC varied between 18% and 52% for patients initially classified under FIGO 2009 Stages IB1 to IIIB. The FIGO 2018 staging system underscores the pivotal role of lymph node metastasis in predicting prognosis and provides valuable insights into the distinct prognostic implications associated with different stages, particularly for early stages. For advanced stages, incorporation of tumor-related factors such as T stage might better elucidate survival differences and guide clinical treatment decisions.

**Protocol registration:**

CRD 42023451793.

## 1 Introduction

Lymph node involvement has been widely recognized as a crucial factor affecting the prognosis of cervical cancer (1). Previous studies have indicated that lymph node involvement is associated with a 30-50% reduction in the 5-year survival rate (2). The assessment of lymph node status involves both preoperative imaging methods and postoperative pathological examinations. Currently two classifications are utilized for the pathological staging of cervical cancer in patients undergoing surgical procedures, the 2018 clinical and radiological FIGO staging system, and the 2010 pathological AJCC system. However, unlike the AJCC(American Joint Committee on Cancer) staging system, the previous FIGO (International Federation of Gynecology and Obstetrics) staging system did not incorporate lymph node involvement into its staging criteria (3).

Since the initial publication of the FIGO classification in 1929, the most recently updated version is FIGO 2018. Staging serves as the cornerstone for evaluating prognosis and guiding treatment decisions. The continual modification of the FIGO staging system aims to comprehensively include factors influencing prognosis, thereby enabling more precise clinical guidance and prognosis prediction. The previous FIGO 2009 cervical cancer system relied solely on clinical examination, while the revised FIGO 2018 system incorporates clinical findings, imaging findings, and pathological information (4). Notably, one significant change in the revised FIGO 2018 system for cervical cancer is the inclusion of lymph node status. Pelvic lymph node and paraaortic lymph node involvement are now defined as IIICI and IIIC2, respectively. Imaging-diagnosed lymph node involvement is defined as IIICr, while pathological confirmation of positive lymph nodes is defined as IIICp.

The rationality of incorporating the Stage IIIC has been the subject of scrutiny in some studies. While certain previous studies found no survival difference when comparing Stage IIIC with IIIA and IIIB, questioning the sole classification of the Stage IIIC (5, 6), others have identified prognostic factors such as the number of positive lymph nodes and tumor size. These studies recommend a more nuanced subtype classification of the Stage IIIC based on these factors (7, 8).

Our study aims to review reports on the stage migration rate to IIIC, allowing us to understand the impact of the new staging system on patient classification compared to the old version. Additionally, we compare the survival rates of different stages with IIIC1, evaluating whether the exclusive classification of IIIC adequately reflects diverse prognoses. Lastly, we synthesize various prognostic factors influencing IIIC1 and explore the possibility of developing a more accurate subcomponent type of IIIC to enhance treatment guidance and prognosis evaluation. Through a meta-analysis of these aspects, we seek to provide valuable insights for guiding clinical treatment.

## 2 Methods

### 2.1 Protocol registration

This systematic review and meta-analysis adhered to the Preferred Reporting Items for Systematic Reviews and Meta-analyses (PRISMA) guidelines and was registered with the International Prospective Register of Systematic Reviews (PROSPERO) (CRD42024501148) (9).

### 2.2 Eligibility criteria

Women who have undergone surgical treatment for cervical cancer will be included in this study. These individuals will be categorized based on the FIGO 2009 staging system and subsequently reclassified using the FIGO 2018 staging system. Exclusion criteria entail patients with synchronous tumor. All potentially eligible studies, including cross-sectional studies, longitudinal cohort studies, case reports, and series case reports published in English, were considered. The inclusion criteria were studies that: (a) investigated the stage migration of patients with cervical cancer from FIGO 2009 to Stage IIIC in the FIGO 2018 Staging System, (b) in survival outcome analysis, provided effect data or enabled the calculation of these data, and (c) if data subsets had been published in more than one article, only the largest sample size was included. The exclusion criteria were: (a) redundant publications, (b) incomplete data, and (c) conference abstracts and reviews.

### 2.3 Search strategy and study selection

PubMed, Embase, Cochrane Central Register of Controlled Trials (CENTRAL), International Clinical Trials Registry Platform (ICTRP), and ClinicalTrials.gov were searched from inception to February 6, 2024. The reference lists of published reviews and retrieved articles were checked for additional trials. Predefined search strings were as follows: “cervical cancer”, “cervical carcinoma”, “cervical squamous cell carcinoma”, “cervical adenocarcinoma”, “nodal involvement”, “nodal metastasis”, “node involvement”, “node metastasis”, “nodes involvement”, “nodes metastasis”, “node positive”, “LN involvement”, “LN metastasis”, “LN positive”, “positive LV”, “positive lymph node”, “positive pelvic lymph node”, “metastatic pelvic lymph nodes”, “metastatic lymph nodes”, “metastatic lymph node”, “FIGO 2018”, “2018 FIGO”, “International Federation of Gynecology and Obstetrics 2018” and “2018 International Federation of Gynecology and Obstetrics”.

Two researchers (HC and LH) independently screened titles and abstracts to assess study eligibility. After initial selection, full texts of potential articles were independently reviewed by two researchers (HC and LH) for further evaluation. Disagreements were resolved through discussion with XT.

### 2.4 Data extraction

Data were extracted by two independent reviewers (HC and YC) in duplicate. A pre-defined extraction table was used to capture data, including the country where the study was located, study quality, and outcomes (the rate of stage migration to IIIC and survival). Double data entry was conducted.

### 2.5 Risk of bias assessment

Two reviewers (LH and YC) independently assessed the quality of included studies. Differences were resolved through discussion, and if no consensus was reached, a third review author (AZ) was involved. Cohort studies included in the prognosis analysis were assessed using the Newcastle–Ottawa Scale (NOS) based on three categories: selected cases, comparability of groups, and assessment of outcomes. Studies awarded six or more stars were classified as having high quality.

### 2.6 Statistical analysis

STATA 16.0 (StataCorp, USA) was used for meta-analysis. Hazard ratios (HRs) with 95% confidence intervals (CIs) were used to combine data on survival outcomes. For studies reporting survival data only in the form of Kaplan-Meier curves, Engauge Digitizer 4.1 was used to extract survival data, and HRs and CIs were calculated following reported methods. The stage migration to IIIC rate (%) was investigated by logistic regression analysis. A p-value of <0.05 was considered statistically significant for the meta-analysis. Heterogeneity between studies was assessed using the I² test: I²<30% indicated low heterogeneity, 30%<I²<50% moderate heterogeneity, and I²≥50% high heterogeneity. Substantial heterogeneity warranted the use of a random-effects model, while a fixed-effects model was applied otherwise. As fewer than 10 articles were included, publication bias analysis was not conducted. Results that could not be meta-analyzed were reported narratively.

## 3 Results

### 3.1 Study selection and characteristics

The study selection process is succinctly outlined in **Supplementary Figure 1**. After removing duplicates, 2922 articles were retrieved and screened based on their titles and abstracts. Subsequently, 46 full texts were obtained for further assessment, of which 21 articles were excluded after a thorough review of the full texts because that it does not align with the final inclusion criteria. Ultimately, the analysis included 25 studies involving 82954 participants (5, 7, 8, 10–31). **Table 1**, **Supplementary Table 1** presents the general characteristics of these studies. All included studies were retrospective and received a NOS rating of six or more stars.

**Table 1:** Characteristics of included studies.

Study	Country	Study type	Number of patients	Median follow-up times	Primary Treatment
Anchora2020 (10)	Italy	Retrospective	541	47(3-302) months	Radical surgery
Alanyali2023 (11)	Istanbul	Retrospective	567	59(3-288) months	Radical surgery
Aslan2019 (12)	Turkey	Retrospective	185	45.5(3-135) months	Radical surgery
Ayhan2019 (13)	Turkey	Retrospective	425	47(3-149) months	Radical surgery
Bogani2019 (14)	Italy	Retrospective	177	58(4-175) months	Radical surgery
Brodeur2021 (15)	Canada	Retrospective	207	44.3(38.4-50.1) months	Definitive chemoradiotherapy
Gregorio 2019 (16)	Germany	Retrospective	265	–	Radical surgery
Grigsby2020 (17)	America	Retrospective	1282	7 years	Radical surgery
Duan2023 (18)	China	Retrospective	9452	–	Radical surgery or definitive chemoradiotherapy
Kaur2022 (19)	India	Retrospective	100	62.1 months	Radical surgery
Li2020 (20)	China	Retrospective	273	34 months	Radical surgery
Li2022 (5)	China	Retrospective	5212	40 months	Radical surgery or Radiotherapy
Liu2020 (21)	China	Retrospective	325	28.4(1.9-114.2) months	Definitive chemoradiotherapy
Long2022 (8)	China	Retrospective	418	–	Radical surgery or definitive chemoradiotherapy
Maeda2023 (22)	Japan	Retrospective	196	50.4 months	Radical surgery or definitive chemoradiotherapy
Matsuo2018 (7)	USA	Retrospective	20642	6.8(3.0-12.2)years	Radical surgery
Mohamud2022 (23)	Denmark	Retrospective	4461	–	Fertility-sparing or radical surgery
Osaku2021 (24)	Japan	Retrospective	153	2112(71-4475) days	Radical surgery
Raut2020 (25)	India	Retrospective	615	33(3-63) months	Definitive chemoradiotherapy
Sert2021 (26)	Turkey	Retrospective	181	61.5(8-132) months	Radical surgery
Shigeta2023 (27)	Japan	Retrospective	1392	–	Radical surgery
Tang2021 (28)	China	Retrospective study	3238	50.1(2-163) months	Radical surgery
Wright2019 (29)	USA	Retrospective study	62212	-	Radical surgery or definitive chemoradiotherapy
Yan2019 (30)	China	Retrospective study	662	68(4-96) months	Radical surgery
Zong2019 (31)	China	Retrospective study	384	43(3-171) months	Radical surgery

### 3.2 Stage migration to IIIC

**Figure 1:**
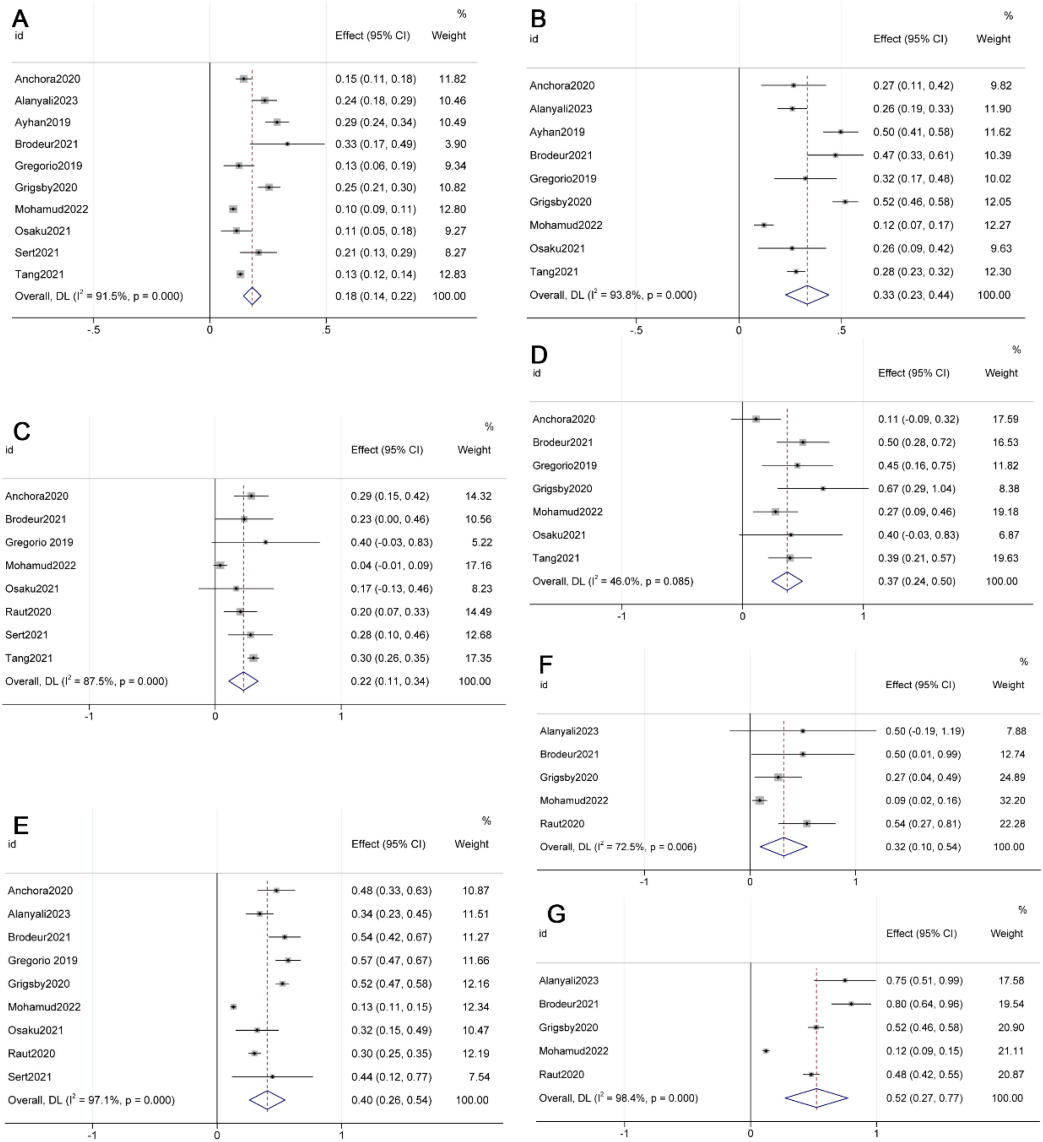
Forest plot illustrating the migration from FIGO 2009 [**(A)** IB1; **(B)** IB2; **(C)** IIA1; **(D)** IIA2; **(E)** IIB; **(F)** IIIA; **(G)** IIIB] to FIGO 2018 stage IIIC.

The meta-analysis results revealed that 18% (95% CI 0.14-0.22, p=0.000, I²=91.5%) of FIGO 2009 Stage IB1 patients were reclassified as Stage IIIC in FIGO 2018 (See **Figure 1**) (10, 11, 13, 15–17, 23, 24, 26, 28). Additionally, the proportions for other stages transitioning to IIIC in FIGO 2018 were as follows: 33% (95% CI 0.23-0.44, p=0.000, I²=93.8%) for Stage IB2, 22% (95% CI 0.11-0.34, p=0.000, I²=87.5%) for IIA1, 37% (95% CI 0.24-0.50, p=0.000, I²=46.0%) for IIA2, 40% (95% CI 0.26-0.54, p=0.000, I²=97.1%) for IIB, 32% (95% CI 0.10-0.54, p=0.005, I²=72.5%) for IIIA, and 52% (95% CI 0.27-0.77, p=0.000, I²=98.4%) for IIIB (see **Figure 1**) (10–12, 15–17, 23–26, 28).

### 3.3 Survival outcomes for stages in FIGO 2018 compared to Stage IIIC1

**Figure 2:**
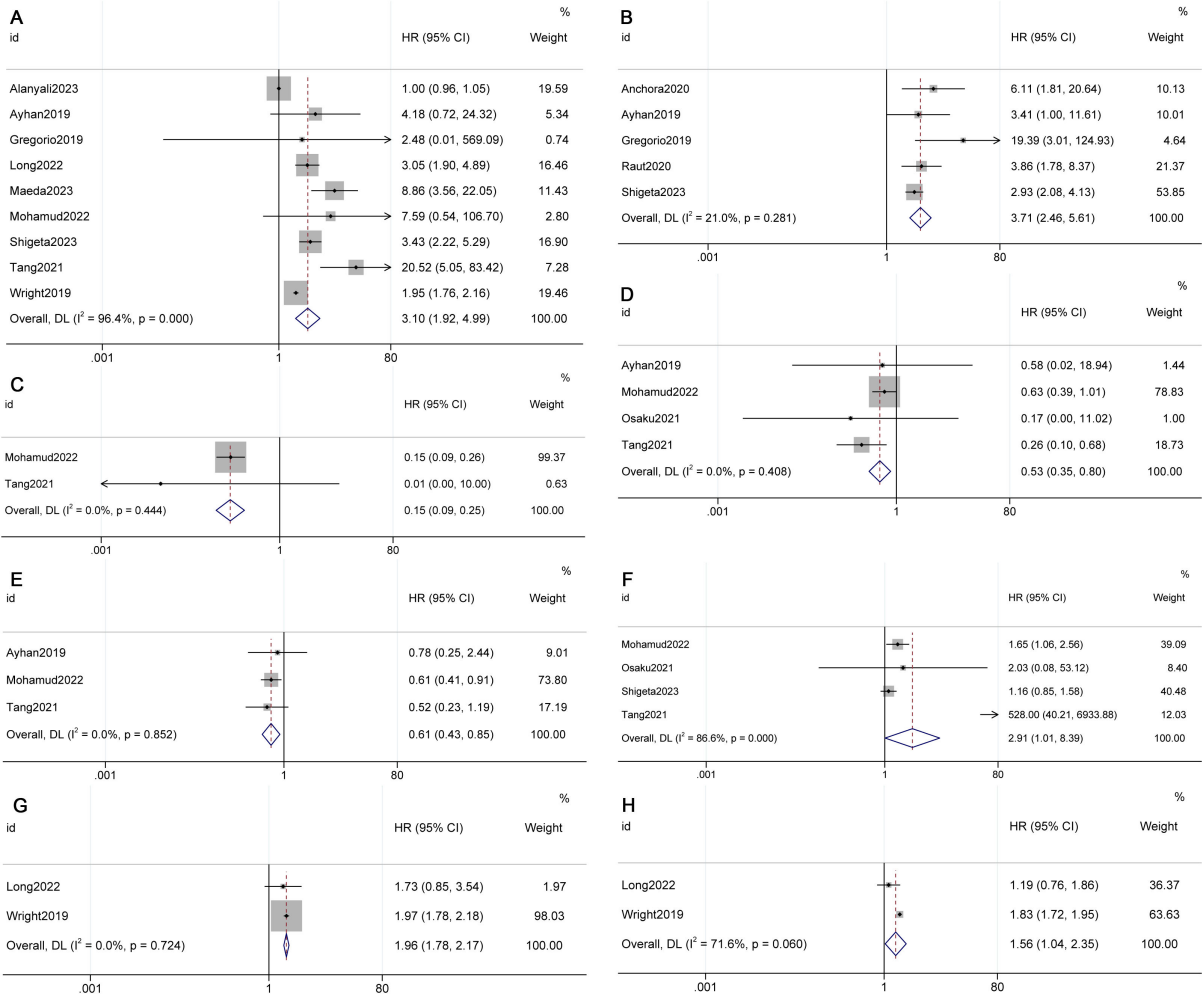
Forest plot presenting pooled survival outcomes for FIGO 2018 stages **(A)** Overall survival (OS) of IIIC2; **(B)** Disease-free survival (DFS) of IIIC2; **(C)** OS of IA1; **(D)** OS of IB1; **(E)** OS of IB2; **(F)** OS of IIB; **(G)** OS IIIA; **(H)** OS of IIIB) compared to stage IIIC1.

The meta-analyses revealed a significant decrease in overall survival (OS) (HR 3.10, 95% CI 1.92-4.99, p=0.000, I²=96.4%) and disease-free survival (DFS) (HR 3.71, 95% CI 2.46-5.61, p=0.017, I²=21.0%) in Stage IIIC2 compared to Stage IIIC1 (see **Figure 2**) (10, 11, 16, 25, 27). However, there was no significant difference in progression-free survival (PFS) between IIIC2 and IIIC1 (HR 2.89, 95% CI 0.34-24.54, p=0.330, I²=0.0%) (see **Supplementary Figure 2**) (11, 22).

The meta-analysis results demonstrated improved OS in Stage IB1 (HR 0.53, 95% CI 0.35-0.80, p=0.003, I²=0.0%) and IB2 (HR 0.61, 95% CI 0.43-0.85, p=0.004, I²=0.0%) compared to Stage IIIC1 (see **Figure 2**) (13, 23, 24, 28). However, there was a decrease in OS in Stage IIB (HR 2.91, 95% CI 1.01-8.39, p=0.047, I²=86.6%), IIIA (HR 1.96, 95% CI 1.78-2.17, p=0.000, I²=0.0%), and IIIB (HR 1.56, 95% CI 1.04-2.35, p=0.031, I²=71.6%) (see **Figure 2**) (8, 23, 24, 27, 28, 29). No significant differences were observed in OS for IB3 (HR 1.35, 95% CI 0.85-2.17, p=0.208, I²=0.0%), and in DFS for IIA (HR 1.60, 95% CI 0.67-3.82, p=0.290, I²=0.0%) and IIB (HR 0.88, 95% CI 0.52-1.51, p=0.649, I²=64.8%) when compared with IIIC1 (see **Supplementary Figure 2**) (10, 13, 19, 23, 25, 27, 28).

### 3.4 Factors affecting survival of Stage IIIC1

#### 3.4.1 Tumor size

**Figure 3:**
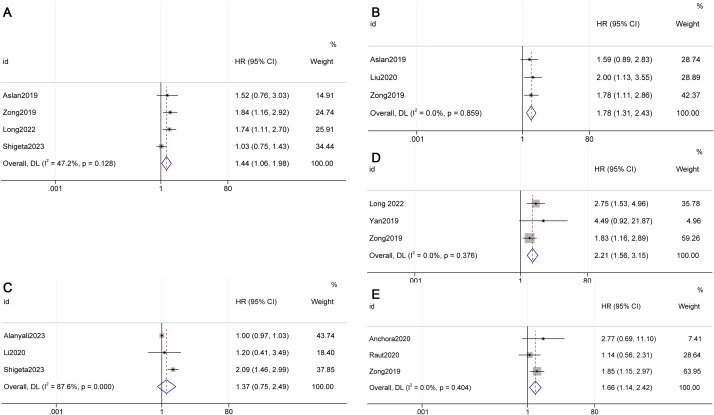
Factors influencing survival in FIGO 2018 stage IIIC1. **(A)** Overall survival (OS) for different tumor sizes (< 4cm vs. ≥ 4cm), **(B)** Disease-free survival (DFS) for different tumor sizes (< 4cm vs. ≥ 4cm), **(C)** OS for different numbers of metastatic lymph nodes (1 vs. >2), **(D)** OS for different numbers of metastatic lymph nodes (2 vs. >3), **(E)** DFS for different numbers of metastatic lymph nodes (2 vs. >3).

The meta-analysis results indicated that OS (HR 1.44, 95% CI 1.06-1.98, p=0.128, I²=47.2%) and DFS (HR 1.78, 95% CI 1.31-2.43, p=0.000, I²=0.0%) were superior in Stage IIIC1 patients with tumors < 4cm compared to those with tumors ≥ 4cm (see **Figure 3**) (8, 13, 21, 27, 31).

#### 3.4.2 Metastatic lymph nodes

The meta-analysis revealed that when the cut-off value was 2, there was no statistically significant difference in OS (HR 1.37, 95% CI 0.75-2.49, p=0.308, I²=87.6%) between individuals with 1 metastatic lymph node and those with 2 or more metastatic lymph nodes in Stage IIIC1. However, when the cut-off value was 3, a poorer OS (HR 2.21, 95% CI 1.56-3.15, p=0.000, I²=0.0%) and DFS (HR 1.66, 95% CI 1.14-2.42, p=0.009, I²=0.0%) were observed in patients with 3 or more metastatic lymph nodes in Stage IIIC1 (see **Figure 3**) (8, 10, 25, 30, 31).

Aslan et al. found through multivariable analyses that lymph node ratio (LNR)≥0.05 were independent prognostic factors for decreased DFS (HR=2.12, 95% CI 1.15–3.90, p=0.015) and OS (HR 1.95, 95% CI 1.01–3.77, p=0.046) in women with 2018 FIGO Stage IIIC cervical cancer (13). Li et al. also found through multivariate analysis that LNR≥0.08 were independent adverse prognostic factors for OS (HR=2.014, 95% CI 1.046–3.875, P=0.036) (5). Bogani et al. found that LNR (HR 20.4, 95% CI 2.39 to 174.9, p=0.006) and the number of positive nodes (HR 1.09, 95% CI 1.05 to 1.14; p<0.001) were correlated with overall survival in univariate analysis; however, only the number of positive nodes (HR 1.06, 95% CI 1.01 to 1.12; p=0.021) correlated with worse survival via multivariate analysis in Stage IIIC tumors (14).

#### 3.4.3 T stage

According to the Tumor Node Metastasis (TNM) Classification based on the American Joint Committee on Cancer Staging System (9th Edition) (32), the T stage for cervical cancer is defined as follows: T1, carcinoma strictly confined to the cervix; T2, carcinoma invading beyond the uterus but not extending to the lower third of the vagina or the pelvic wall; and T3, carcinoma involving the lower third of the vagina and/or extension to the pelvic wall and/or associated hydronephrosis or non-functioning kidney.

Most studies have identified T stage as an independent prognostic factor for survival in Stage IIIC. Li et al. observed significant differences in 5-year OS (100.0%, 81.9%, 76.1%, 74.0%, and 65.0%, p<0.001) and DFS (100.0%, 74.5%, 65.9%, 72.6%, and 61.3%, p<0.001) among the IIIC-T1a, T1b, T2a, T2b, and T3 groups. Multivariate survival analysis revealed that the T1a had no significant correlation with 5-year OS (HR 0.923, P<0.001) or DFS (HR 0.923, P=0.001), while T2a (for OS: HR 1.405, 95% CI 1.076–1.834, p=0.012; for DFS: HR 1.372, 95% CI 1.108–1.699, p=0.004), T2b (for OS: HR 1.592, 95% CI 1.203–2.108, p=0.001; for DFS, HR 1.337, 95% CI 1.061–1.684, p=0.014), and T3 (for OS: HR 2.495, 95% CI 1.971–3.157, p<0.001; for DFS: HR 2.015, 95% CI 1.659–2.446, p<0.001) were associated with lower 5-year OS and DFS (5). Grigsby et al. found a 5-year PFS of 72%, 63%, and 41% for IIIC1-T1, T2, and T3, respectively (p < 0.0001). Similarly, the PFS for IIIC2-T1, T2, and T3 was 62%, 32%, and 23%, respectively (p=0.01). However, the results were not always consistent (17). Raut et al. found that higher T stage had a tendency to have a poorer prognosis (3-year DFS of 81.4% for IIIC1-T1, 64.7% for IIIC1-T2, and 62.7% IIIC1-T3; 100% for IIIC2-T1, 69.2% for IIIC2-T2, and 44.6% for IIIC2-T3); however, this did not reach statistical significance (p>0.05) (25).

Duan et al. reported that compared with IIIC-T3, IIIC-T1/T2 and IIB had a lower risk of death and recurrence. However, there was no significant difference in the risk of death and recurrence between IIIC-T1/T2 and IIB, and between IIIC-T3 and IIIA+IIIB (18). Matsuo et al. found that survival of Stage IIIC1-T3b was significantly poorer compared to those with Stage IIIB (38.1% versus 42.6%, HR 1.12, 95% CI 1.02–1.22, p=0.013) (7). Contrastingly, women with Stage IIIC1-T3a exhibited similar survival rates when compared to those with Stage IIIA (42.9% versus 45.9%, HR 1.01, 95% CI 0.85–1.22, p=0.88). Long et al. observed that the 5-year OS rates in the IIIC1-T1, T2, and T3 were 72.2%, 54.1%, and 18.6%, respectively (p<0.001). The 5-year OS rate was higher in patients with Stage IIIC1-T1 than in those with Stage IIIA (p=0.004) or IIIB (p<0.001) (8).

## 4 Discussion

With the exception of the ESGO (European Society of Gynecological Oncology) guidelines, which are based on the AJCC system, most of the guidelines present their recommendations based primarily on FIGO staging (33). Meanwhile, the American NCCN guidelines incorporate both systems, with a predominant emphasis on FIGO (33). Accumulated evidence from previous studies underscores the significance of lymph node metastasis as a crucial prognostic factor for cervical cancer in FIGO staging system. The integration of positive lymph nodes into the FIGO 2018 staging system led to the reclassification of some early-stage cancers to Stage IIIC. Currently, it has been suggested that there is no difference in prognosis between simple hysterectomy and radical hysterectomy for early-stage cervical cancer (34, 35). In this context, isolated lymph node staging becomes very important. In this meta-analysis, migration rates to FIGO 2018 Stage IIIC ranged from 18% to 37% for FIGO 2009 Stage IB to IIA, surpassing rates reported in earlier studies where lymph node metastasis ranged from 8% to 32% in the early stages of cervical cancers (36, 37). Furthermore, this review revealed that in FIGO 2009 Stage IIB and beyond, the lymph node positivity rate was even higher than in early stage, with migration rates ranging from 32% to 52% to FIGO 2018 Stage IIIC.

This review found that the OS for Stage IIIC1 is inferior to that of Stage IB1 and IB2, comparable to Stage IB3, yet superior to Stage IIB, IIIA, and IIIB. These findings underscore the pivotal role of lymph node metastasis in predicting prognosis and provide valuable insights into the distinct prognostic implications associated with different stages, especially for early stages. Following this guideline and the FIGO 2018 staging, some early-stage (IB-IIA) cervical cancers classified under FIGO 2009 may necessitate a modification in treatment plans due to the reclassification to IIIC. Some authors have advocated for primary chemoradiotherapy to reduce complications associated with surgery combined with postoperative adjuvant chemoradiotherapy (38, 39). Others have proposed postoperative adjuvant chemoradiotherapy following surgery, as it may lead to higher survival rates and reduced pelvic recurrence (37, 40). Stage migration may lead to a shift in therapeutic approaches (surgery versus exclusive radio-chemotherapy), potentially impacting the quality of life and sexual function of women with cervical cancer (41). Future research could explore the optimal treatment approaches for these patients in the new FIGO staging.

Unlike early-stage tumors, there was no significant difference in the OS for advanced-stage tumors after restaging according to FIGO 2018 system. This could be because factors other than positive lymph nodes, such as parametrial involvement and large tumor size, also play crucial roles in prognosis. Hence, some critics questioned the rationale behind the solitary categorization of IIIC for advanced stages. However, we believe that although Stage IIIC may not reflect differences in survival in advanced tumors, the new staging does not alter the treatment plan for these patients. Additionally, patients diagnosed with Stage IIIC2 exhibit distinct survival outcomes compared to IIIC1, highlighting the effectiveness of the FIGO 2018 system in capturing variations in survival outcomes between pelvic and paraaortic lymph nodes, ensuring precision in staging.

Since the introduction of the new FIGO staging, substantial research attention has been devoted to refining the subgroup analysis of Stage IIIC, aiming to better elucidate staging advantages. Prior studies identified a tumor size exceeding 4 cm as a prognostic factor for Stage IIIC1 (21, 22, 31). This meta-analysis also found a significant survival difference at the 4 cm threshold in Stage IIIC1. Given this survival difference, there is a growing consensus to incorporate tumor size as an additional consideration for risk stratification in Stage IIIC1.

The impact of the number of metastatic lymph nodes on survival has been explored in previous studies, with varying cutoff values between 1-5 (20). However, controversy surrounds its effect on the survival rate of IIIC1, as some studies argue it is not a prognostic factor (20, 25), while others assert its significance (10, 21). This study indicated that patients with 3 or more metastatic lymph nodes in Stage IIIC1 exhibited differences in both DFS and OS. The lymph node ratio (LNR), defined as the number of metastatic lymph nodes divided by the total number of lymph nodes harvested, emerged as a predictor for the prognosis of Stage IIIC (12). Some studies reported that LNR was associated with the prognosis and recommended LNR≥0.1, 0.08, 0.05 differently as the cut off value for prognosis (5, 11, 12). However, Bogani et al. failed to found LNR was the factor correlated with worse survival in multifactor analysis (14). The lack of a uniform cutoff and controversy over its relationship with survival outcomes necessitate further exploration.

Most included studies found that the survival outcomes of Stage IIIC1 were associated with the latest American Joint Committee on Cancer TNM staging system and identified T stage as an independent prognostic factor for survival outcomes (7, 8, 17, 25). The diverse survival outcomes observed in Stage IIIC1 underscore that prognosis may be influenced by a combination of both lymph node and tumor-related factors. The inclusion of earlier T stage cervical cancer migration contributes to the favorable survival outcome observed in IIIC1. Duan et al. proposed categorizing Stage IIIC-T1, T2 as Stage IIC, distinct from Stage IIIC-T3a, T3b, due to differential treatment plans and prognosis (18). Given these findings, the incorporation of T stage for risk stratification in Stage IIIC1 could better delineate survival differences and guide clinical treatment decisions.

Despite following a rigorous review protocol for study selection, data extraction, and analysis, this study has some limitations. The retrospective nature of included studies inherently carries limitations. Some subgroup analyses lacked uniform standards, precluding meta-analysis. Additionally, the use of a random-effects model may introduce variability in weighting large studies during statistical heterogeneity, impacting the combined results. We have utilized both random-effects and fixed-effect models based on the level of heterogeneity observed in our data. This approach allows us to more accurately capture the variability in the results across different studies. These limitations warrant cautious interpretation of the study’s findings.

In summary, the migration rates to FIGO 2018 Stage IIIC varied between 18% and 52% for patients initially classified under FIGO 2009 Stages IB1 to IIIB. The FIGO 2018 staging system underscores the pivotal role of lymph node metastasis in predicting prognosis and provides valuable insights into the distinct prognostic implications associated with different stages, particularly for early stages. For advanced stages, incorporation of tumor-related factors such as T stage might better elucidate survival differences and guide clinical treatment decisions.

## Supplementary material

Supplementary Table 1 The detailed information of the stage migration.

Supplementary Figure 1 Study Selection Flowchart.

Supplementary Figure 2 Forest plot depicting pooled survival outcomes for stages in FIGO 2018 (**(A)** Progression-free survival (PFS) of IIIC2; **(B)** Overall survival (OS) of IA2; **(C)**. OS of IB3; **(D)** DFS of IIA; **(E)** DFS of IIB) compared to stage IIIC1.
