# Supplementary material for: Stage migration and survival outcomes in patients with cervical cancer at Stage IIIC according to the 2018 FIGO staging system: a systematic review and meta-analysis
Source: Front Oncol. 2024 Oct 1;14:1460543. doi: 10.3389/fonc.2024.1460543 (PMC11473289; doi:10.3389/fonc.2024.1460543)
Supplement: Supplementary Table 1 — The detailed information of the stage migration. [file DataSheet1.docx]

Additional Table 1

| Study | Stage migration | | | | | | | | | | | | | | | | | |
| --- | --- | --- | --- | --- | --- | --- | --- | --- | --- | --- | --- | --- | --- | --- | --- | --- | --- | --- |
|  | IA_1_ | IIIC | IA_2_ | IIIC | IB_1_ | IIIC | IB_2_ | IIIC | IIA_1_ | IIIC | IIA_2_ | IIIC | IIB | IIIC | IIIA | IIIC | IIIB | IIIC |
| Anchora2020[10] | - | - | - | - | 418 | 61 | 30 | 8 | 42 | 12 | 9 | 1 | 42 | 20 | - | - | - | - |
| Alanyali2022[11] | 2 | 0 | 5 | 2 | 254 | 60 | 146 | 38 | - | - | - | - | 70 | 24 | 2 | 1 | 12 | 9 |
| Aslan2019[12] | - | - | - | - | - | - | - | - | - | - | - | - | - | - | - | - | - | - |
| Ayhan2019[13] | - | - | - | - | 294 | 85 | 131 | 65 | - | - | - | - | - | - | - | - | - | - |
| Bogani2019[14] | - | - | - | - | - | - | - | - | - | - | - | - | - | - | - | - | - | - |
| Brodeur2021[15] | 2 | 2 |  |  | 33 | 11 | 51 | 24 | 13 | 3 | 20 | 10 | 59 | 32 | 4 | 2 | 25 | 20 |
| Gregorio 2019[16] | - | - | - | - | 96 | 12 | 37 | 12 | 5 | 2 | 11 | 5 | 93 | 53 | - | - | - | - |
| Grigsby2020[17] | 9 | 1 | 15 | 1 | 322 | 82 | 243 | 126 | 8 | 0 | 6 | 4 | 328 | 172 | 15 | 4 | 248 | 129 |
| Duan2023[18] | - | - | - | - | - | - | - | - | - | - | - | - | - | - | - | - | - | - |
| Kaur2022[19] | - | - | - | - | - | - | - | - | 4 | 0 | - | - | - | - | - | - | - | - |
| Li2020[20] | - | - | - | - | - | - | - | - | - | - | - | - | - | - | - | - | - | - |
| Li2022[5] | - | - | - | - | - | - | - | - | - | - | - | - | - | - | - | - | - | - |
| Liu2020[21] | - | - | - | - | - | - | - | - | - | - | - | - | - | - | - | - | - | - |
| Long2022[8] | - | - | - | - | - | - | - | - | - | - | - | - | - | - | - | - | - | - |
| Maeda2023[22] | - | - | - | - | - | - | - | - | - | - | - | - | - | - | - | - | - | - |
| Matsuo2018[7] | - | - | - | - | - | - | - | - | - | - | - | - | - | - | - | - | - | - |
| Mohamud2022[23] | 827 | 2 | 62 | 0 | 1670 | 167 | 181 | 22 | 50 | 2 | 22 | 6 | 871 | 114 | 66 | 6 | 423 | 52 |
| Osaku2021[24] | - | - | - | - | 87 | 10 | 27 | 7 | 6 | 1 | 5 | 2 | 28 | 9 | - | - | - | - |
| Raut2020[25] | - | - | - | - | - | - | - | - | 35 | 7 | - | - | 317 | 95 | 13 | 7 | 218 | 105 |
| Sert2021[26] | - | - | 4 | 1 | 100 | 21 | 42 | 0 | 25 | 7 | - | - | 9 | 4 | - | - | 1 | 0 |
| Shigeta2023[27] | - | - | - | - | - | - | - | - | - | - | - | - | - | - | - | - | - | - |
| Tang2021[28] | 12 | 0 | 71 | 1 | 2361 | 310 | 380 | 106 | 386 | 117 | 28 | 11 | - | - | - | - | - | - |
| Wright2019[29] | - | - | - | - | - | - | - | - | - | - | - | - | - | - | - | - | - | - |
| Yan2019[30] | - | - | - | - | - | - | - | - | - | - | - | - | - | - | - | - | - | - |
| Zong2019[31] | - | - | - | - | - | - | - | - | - | - | - | - | - | - | - | - | - | - |
