# Supplementary figures and images for: Stage migration and survival outcomes in patients with cervical cancer at Stage IIIC according to the 2018 FIGO staging system: a systematic review and meta-analysis
Source: Front Oncol. 2024 Oct 1;14:1460543. doi: 10.3389/fonc.2024.1460543 (PMC11473289; doi:10.3389/fonc.2024.1460543)

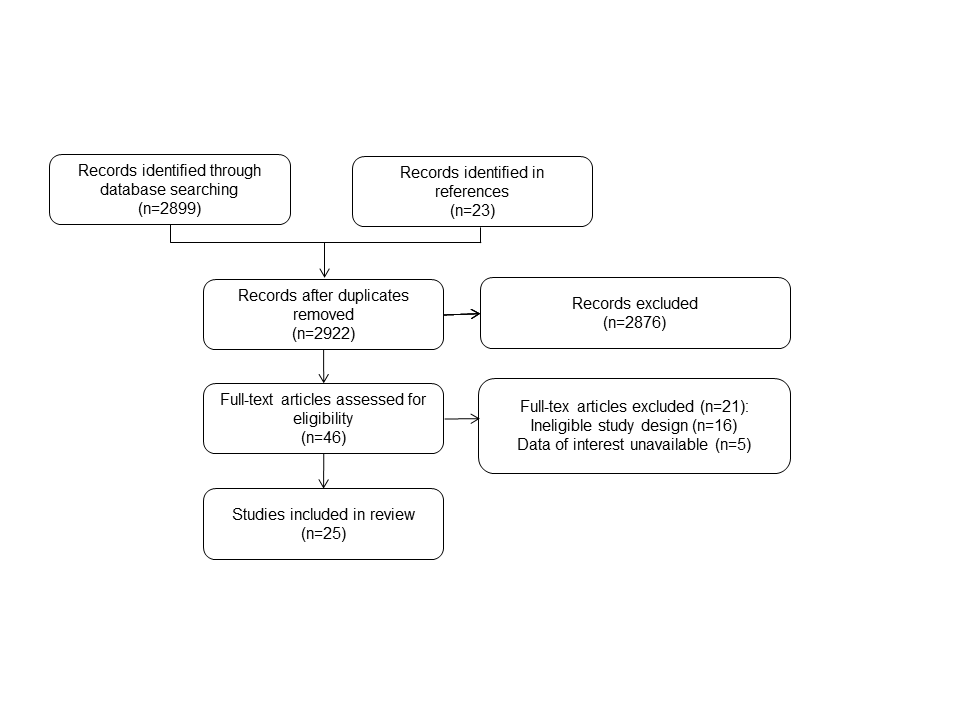

Supplement: Supplementary Figure 1 — Study Selection Flowchart. [file Image1.tif]

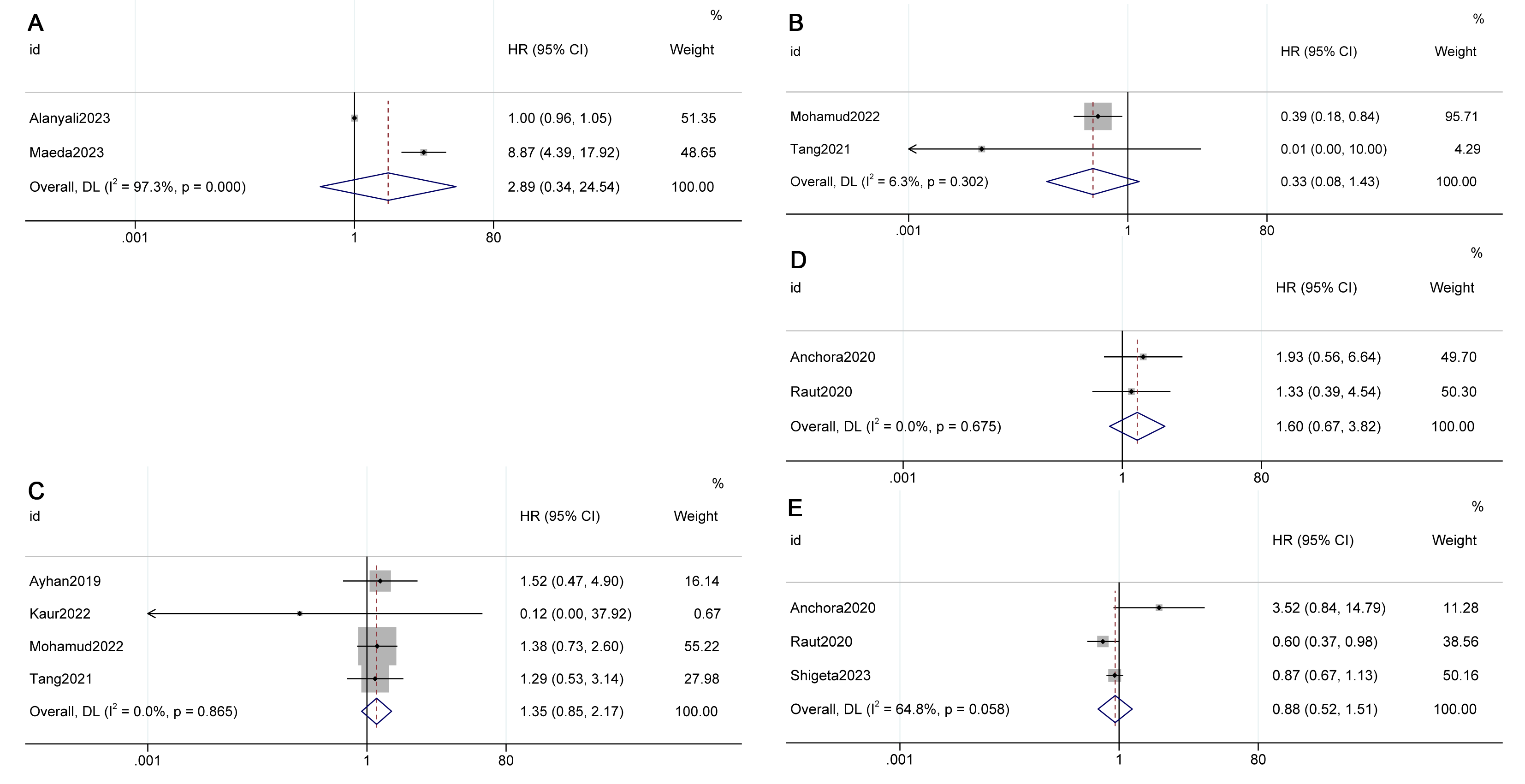

Supplement: Supplementary Figure 2 — Forest plot depicting pooled survival outcomes for stages in FIGO 2018 ((A) Progression-free survival (PFS) of IIIC2; (B) Overall survival (OS) of IA2; (C). OS of IB3; (D) DFS of IIA; (E) DFS of IIB) compared to stage IIIC1. [file Image2.tif]
